# Biodistribution and internal radiation dosimetry of a companion diagnostic radiopharmaceutical, [^68^Ga]PSMA-11, in subcutaneous prostate cancer xenograft model mice

**DOI:** 10.1038/s41598-021-94684-6

**Published:** 2021-07-27

**Authors:** Su Bin Kim, In Ho Song, Yoo Sung Song, Byung Chul Lee, Arun Gupta, Jae Sung Lee, Hyun Soo Park, Sang Eun Kim

**Affiliations:** 1 Department of Applied Bioengineering, Graduate School of Convergence Science and Technology, Seoul National University, 1 Gwanak-ro, Gwanak-gu, Seoul, 08826 Korea; 2 Department of Nuclear Medicine, Seoul National University College of Medicine, Seoul National University Bundang Hospital, 82 Gumi-ro, 173 Beon-gil, Bundang-gu, Seongnam, 13620 Korea; 3 Advanced Institutes of Convergence Technology, 145 Gwanggyo-ro, Yeongtong-gu, Suwon, 16229 Korea; 4 Department of Radiology and Imaging Institution: B.P. Koirala Institute of Health Sciences (BPKIHS), Dharan-18, Province-1, Sunsari, Nepal; 5 Department of Nuclear Medicine, Seoul National University College of Medicine, 103 Daehak-ro, Jongno-gu, Seoul, 03080 Korea; 6 Department of Molecular Medicine and Biopharmaceutical Sciences, Graduate School of Convergence Science and Technology, Seoul National University, 1 Gwanak-ro, Gwanak-gu, Seoul, 08826 Korea

**Keywords:** Positron-emission tomography, Cancer imaging

## Abstract

[^68^Ga]PSMA-11 is a prostate-specific membrane antigen (PSMA)-targeting radiopharmaceutical for diagnostic PET imaging. Its application can be extended to targeted radionuclide therapy (TRT). In this study, we characterize the biodistribution and pharmacokinetics of [^68^Ga]PSMA-11 in PSMA-positive and negative (22Rv1 and PC3, respectively) tumor-bearing mice and subsequently estimated its internal radiation dosimetry via voxel-level dosimetry using a dedicated Monte Carlo simulation to evaluate the absorbed dose in the tumor directly. Consequently, this approach overcomes the drawbacks of the conventional organ-level (or phantom-based) method. The kidneys and urinary bladder both showed substantial accumulation of [^68^Ga]PSMA-11 without exhibiting a washout phase during the study. For the tumor, a peak concentration of 4.5 ± 0.7 %ID/g occurred 90 min after [^68^Ga]PSMA-11 injection. The voxel- and organ-level methods both determined that the highest absorbed dose occurred in the kidneys (0.209 ± 0.005 Gy/MBq and 0.492 ± 0.059 Gy/MBq, respectively). Using voxel-level dosimetry, the absorbed dose in the tumor was estimated as 0.024 ± 0.003 Gy/MBq. The biodistribution and pharmacokinetics of [^68^Ga]PSMA-11 in various organs of subcutaneous prostate cancer xenograft model mice were consistent with reported data for prostate cancer patients. Therefore, our data supports the use of voxel-level dosimetry in TRT to deliver personalized dosimetry considering patient-specific heterogeneous tissue compositions and activity distributions.

## Introduction

Prostate cancer is one of the most common malignancies and is the leading cause of cancer-related deaths in men^1^. Prostate-specific membrane antigen (PSMA) is a cell surface protein that exhibits a significantly increased expression in prostate cancer cells, which makes it ideally suited for molecular imaging^2^. Having demonstrated remarkable affinity to PSMA in numerous trials, [^68^Ga]PSMA-11 has already been adopted for clinical use at several institutions worldwide and has become established as the most widely used diagnostic radiopharmaceutical for positron emission tomography (PET) in clinical practice. With the ability to define the location and extent of disease, PET imaging with [^68^Ga]PSMA-11 has been regarded as a significant step forward in the diagnosis of prostate cancer^3–5^. Additionally, [^68^Ga]PSMA-11 has been widely demonstrated to be a useful component in the work-up of prostate cancer patients^6,7^. More recently, various PSMA-targeting radioligands that can be labeled with alpha- (e.g., actinium-225) and beta-emitting radionuclide (e.g., lutetium-177 and yttrium-90) have been developed for therapeutic use alongside [^68^Ga]PSMA-11 in terms of companion diagnostics.

The use of PSMA-targeting radiopharmaceuticals for companion diagnostics has gained significant attention during the last decade, coinciding with the promise shown by PET imaging with [^68^Ga]PSMA-11 as a predictor of the response to therapeutic treatment following radionuclide therapy using alpha- (especially with [^225^Ac]PSMA-617) and/or beta-emitting radioisotope-labeled PSMA-ligands. Although the application of [^68^Ga]PSMA-11 PET coupled with therapeutic radiopharmaceuticals is currently under broad clinical and scientific investigation, insufficient preclinical studies have been performed to determine the dosing regimen for the therapeutic radiopharmaceuticals. Owing to the need to estimate the absorbed dose–response relationship for therapeutic radiopharmaceuticals for clinical use, PET is increasingly used to characterize the biodistribution, pharmacokinetics, and internal radiation dosimetry of novel companion diagnostic or theranostic radiopharmaceuticals in disease/target-specific xenograft animal models.

Internal radiation dosimetry has become more important in recent years because of the growing interest in personalized medicine and targeted radionuclide therapy (TRT)^8^. Clinically approved methods for absorbed dose estimation are recommended by the Medical Internal Radiation Dose (MIRD) Committee. Although the MIRD method uses a generalized formalism for estimating the absorbed dose, this approach does not incorporate patient-specific activity distributions and organ anatomies because the standardized geometry is not robust enough to model the size, shape, and location of every unique tumor discovered in patients. As this method calculates the average absorbed dose in each organ, there is a limitation in that the maximum and minimum radiation dose cannot be known, and the dose delivered to the abnormal organ cannot be obtained. Alternatively, voxel-based dosimetry, which implements the Monte Carlo approach to simulate the complete events engaged in the radioactivity decay process, is considered potentially more accurate than the MIRD method because it considers activity distributions, organ anatomies, and tumor tissue heterogeneity on a subject-by-subject basis^9,10^.

In this study, we characterized the biodistribution and pharmacokinetics of [^68^Ga]PSMA-11 in subcutaneous prostate cancer xenograft model for mice and estimated its internal radiation dosimetry for various organs (including the tumor). Our characterization is based on the voxel‐based dosimetry method, which overcomes the drawbacks of the conventional organ-level (or phantom-based) method by evaluating the absorbed dose in the tumor directly using a dedicated Monte Carlo simulation. For comparative purposes, the absorbed dose was also calculated using the MIRD-recommended organ-level method.

## Results

### Biodistribution and pharmacokinetics

**Figure 1:**
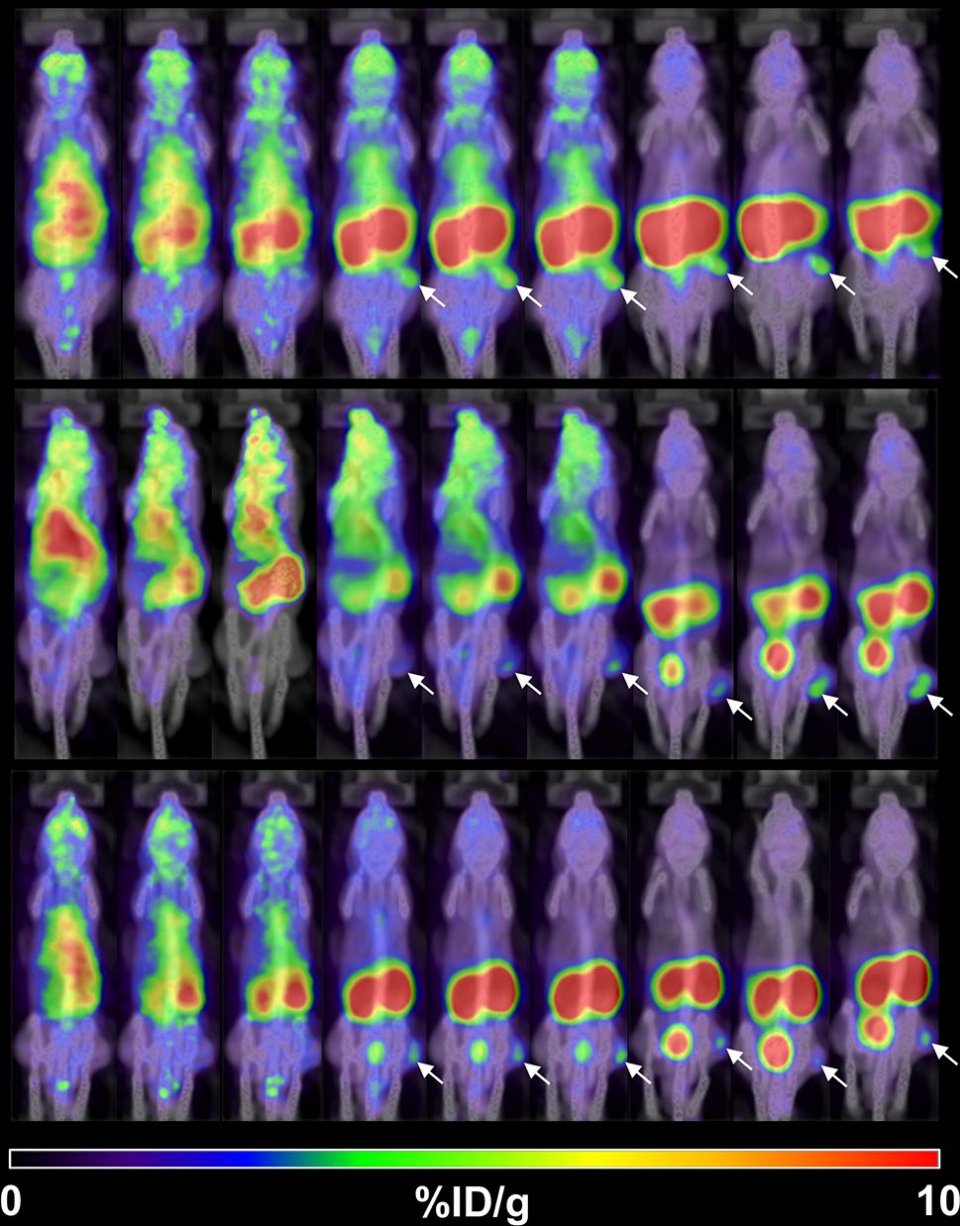
PET/CT images of the subcutaneous prostate cancer xenograft model mice (*n* = 3, each row) at various time-points (from left to right in each column: 2, 5, 10, 30, 60, 90, 180, 240, 300 min p.i., respectively) after the intravenous injection of [^68^Ga]PSMA-11. The arrows indicate the PSMA-positive tumor (22Rv1). %ID/g, percent injected dose per gram of tissue.

**Figure 2:**
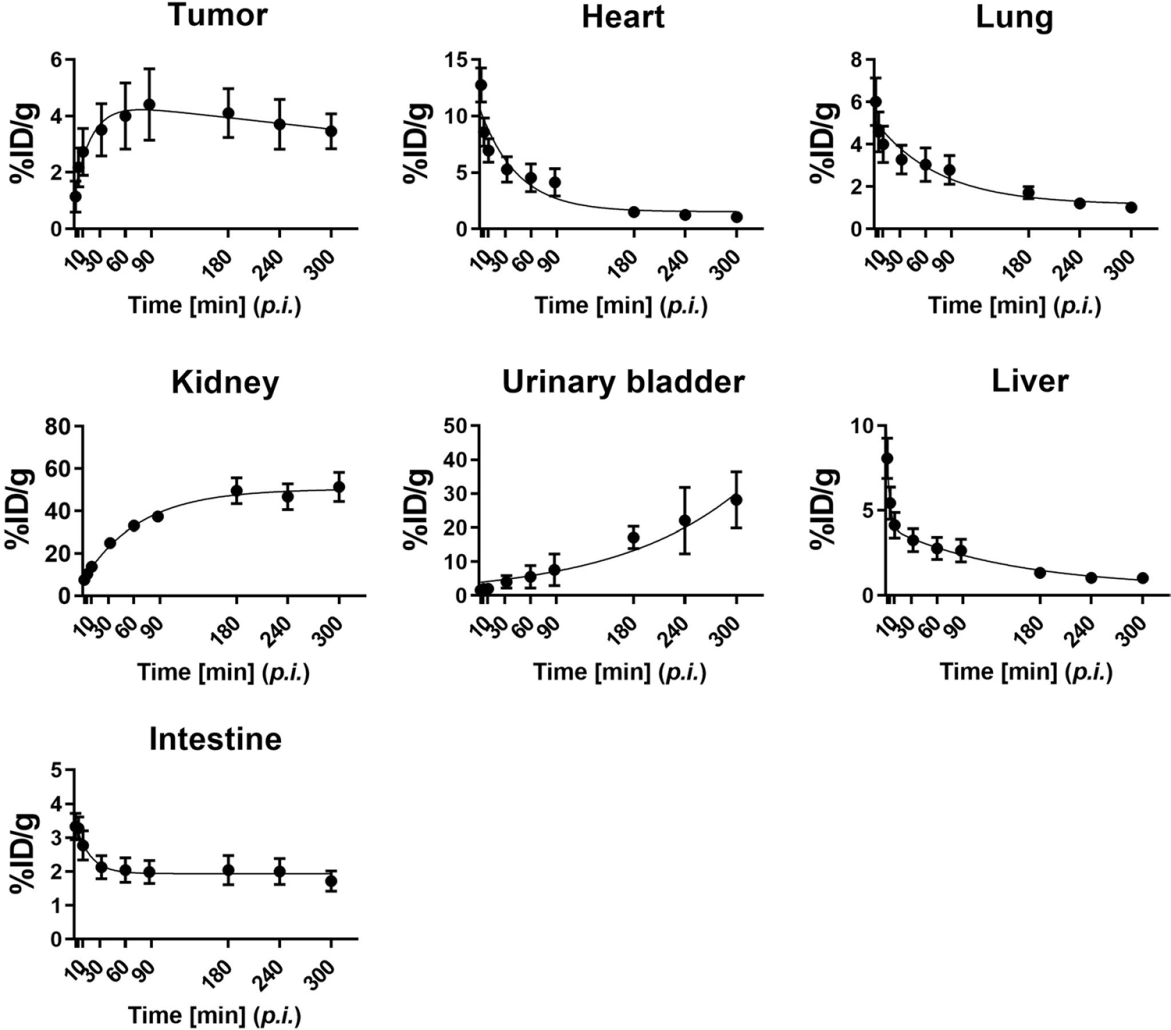
Time courses of [^68^Ga]PSMA-11 distribution in the PSMA-positive tumor (22Rv1) and various organs. The solid lines represent the non-linear least-squares fitted optimization results for the association or dissociation model. The measurement points represent the mean ± the standard error of the mean (SEM; *n* = 3).

Figure 1 shows the biodistribution and clearance of [^68^Ga]PSMA-11 for PSMA-positive tumor (22Rv1)-bearing mice after intravenous injection. The figure illustrates rapid whole-body distribution immediately after the injection, followed by rapid washout (at variable rates) for peripheral organs, including the liver, whereas other organs, namely the kidneys, urinary bladder, and the tumor, demonstrated a longer lasting substantial uptake of [^68^Ga]PSMA-11. The pharmacokinetic parameters for the visualized organs and the tumor are summarized in Fig. 2 and Table 1. The kidneys showed the highest accumulation of [^68^Ga]PSMA-11 without exhibiting a washout phase during the study. The urinary bladder was the predominant excretion route of the intravenous injection of [^68^Ga]PSMA-11 with twofold greater accumulation than the intestine. The tumor exhibited a peak [^68^Ga]PSMA-11 concentration of 4.5 ± 0.7 %ID/g 2 h (on average) after the injection, which decreased gradually thereafter, measuring approximately 3 %ID/g after 5 h. The data demonstrates that the preclinical biodistribution and pharmacokinetics were consistent with previously reported results for prostate cancer patients, particularly in terms of excretion^11^.

**Table 1:** Pharmacokinetic parameters of [^68^Ga]PSMA-11.

Organ	T_max_ [min]	C_max_ [%ID/g]	AUC [%ID/g min]	T_1/2_ [min]
Tumor	118.67 ± 30.67	4.51 ± 0.69	1153.4 ± 148.7	708.2
Heart	0.58 ± 0.00	30.73 ± 4.85	906.4 ± 196.9	30.24
Lung	0.58 ± 0.00	12.76 ± 3.33	658.9 ± 137.0	51.93
Kidneys	260 ± 40.00	51.97 ± 6.43	12,168.7 ± 819.2	Accumulated
Urinary bladder	260 ± 40.00	28.65 ± 7.81	4197.0 ± 1414.3	Accumulated
Liver	2.14 ± 0.31	8.64 ± 1.23	606.5 ± 102.8	100.5
Intestine	1.64 ± 0.56	4.04 ± 0.27	614.5 ± 108.0	11.6

Values are the mean ± SEM (*n* = 3).

Additionally, the pharmacokinetics of [^68^Ga]PSMA-11 were characterized in terms of selectivity and specificity. In the model mice bearing both PSMA-positive (22Rv1) and negative (PC3) tumors, the uptake of [^68^Ga]PSMA-11 in the 22Rv1 tumor (2.7 ± 0.3 %ID/g) was six-fold greater than the PC3 tumor (0.5 ± 0.1 %ID/g), demonstrating that [^68^Ga]PSMA-11 bound selectively to the PSMA-positive, PSMA-rich tumors (*P* = 0.0030, *t*(*df*) = 6.430(4)). In model mice bearing only 22Rv1 tumors, the inhibition of [^68^Ga]PSMA-11 uptake was significant before and after 2-(Phosphonomethyl)-pentanedioic acid (2-PMPA) (50 mg/kg) treatment, measuring -57.8 ± 15.9 %ID/g (*P* = 0.0485, *t*(*df*) = 2.806 (4)), thereby demonstrating that [^68^Ga]PSMA-11 bound specifically to the PSMA-positive tumors.

### Internal radiation dosimetry

**Figure 3:**
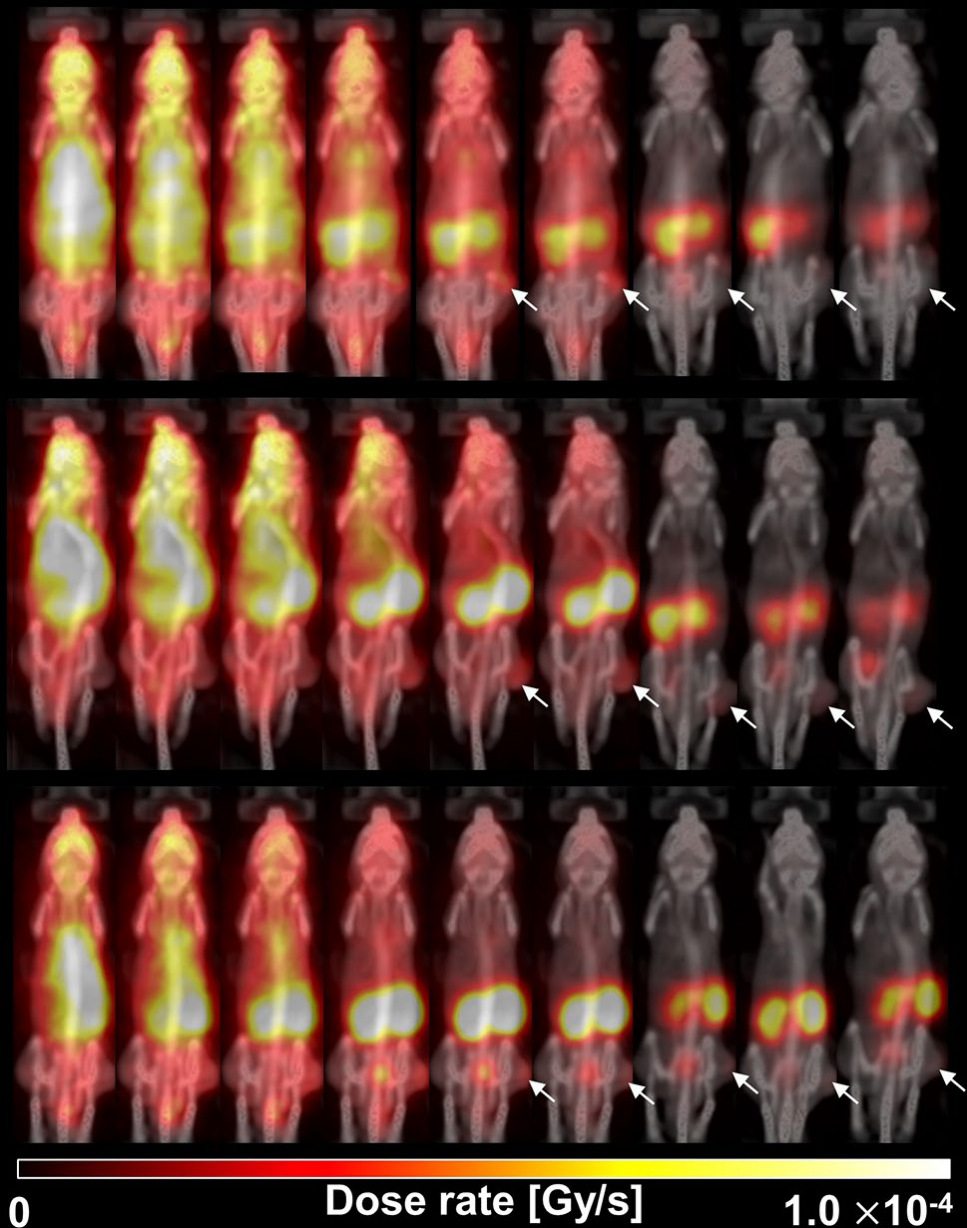
Dose map of [^68^Ga]PSMA-11 in the subcutaneous prostate cancer xenograft model mice (*n* = 3, each row) at various time-points (from left to right in each column: 2, 5, 10, 30, 60, 90, 180, 240, 300 min p.i., respectively) after the intravenous injection of [^68^Ga]PSMA-11. The arrows indicate the PSMA-positive tumor (22Rv1).

**Figure 4:**
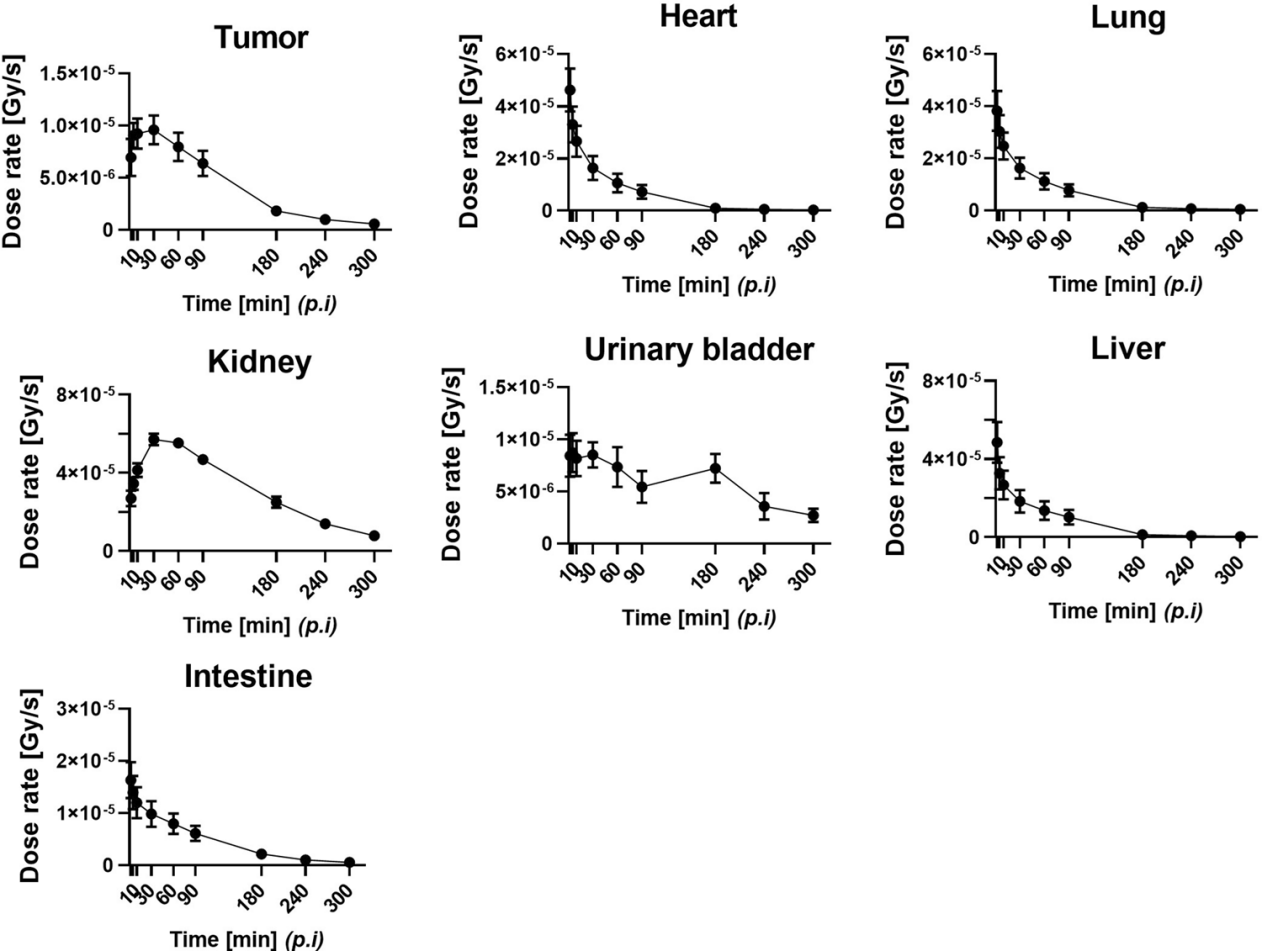
Dose rate curves of [^68^Ga]PSMA-11 in the PSMA-positive tumor (22Rv1) and various organs. Values are the mean ± SEM (*n* = 3).

The Monte Carlo simulation successfully generated the corresponding dose distribution maps for each voxelized source—i.e., the [^68^Ga]PSMA-11 PET images (uncorrected for radiation decay) of every time-point, which represent the amount of radioactive decay at given time-point in the simulation of interaction between particles and materials—against the voxelized phantom—i.e., the CT images of every time-point, which represent materials such as air, air-body interface, soft tissue, and bone that were segmented according to the threshold of the Hounsfield unit values used in the simulation—of each individual model mouse (Fig. 3). Because the voxel values, which indicate the temporal changes in dose (dose-rate) at each time-point, in the dose maps are expressed in Gy/s, organ specific absorbed doses are represented by dividing the integral sum of the area under the dose rate curve (Fig. 4) for each organ by the administered radioactivity. The calculated absorbed doses are summarized in Table 2, along with the results estimated using the MIRD-recommended organ-level method.

**Table 2:** Absorbed dose received by organs of the subcutaneous prostate cancer xenograft model mice after [^68^Ga]PSMA-11 administration.

Organ	Absorbed dose (Gy/MBq)		Difference (organ-level - voxel-level)	*P*
	Voxel-level	Organ-level		
Tumor	0.024 ± 0.003	N/A	N/A	N/A
Heart	0.034 ± 0.009	0.055 ± 0.010	0.021 ± 0.004	0.0225
Lung	0.035 ± 0.008	0.045 ± 0.009	0.009 ± 0.001	0.4845
Kidney	0.209 ± 0.005	0.492 ± 0.059	0.283 ± 0.055	0.0088
Urinary bladder	0.038 ± 0.010	0.451 ± 0.199	0.413 ± 0.188	0.1065
Liver	0.041 ± 0.012	0.025 ± 0.002	−0.016 ± 0.010	0.2186
Intestine	0.026 ± 0.004	0.013 ± 0.000	−0.014 ± 0.003	0.0227

Values are the mean ± SEM (*n* = 3).

By inspecting voxel levels, it is observed that the level of [^68^Ga]PSMA-11 accumulation corresponds to the level of the absorbed dose in each organ. The absorbed dose was the highest in the kidneys (0.209 ± 0.005 Gy/MBq), followed by the liver, urinary bladder and lungs. The variance in the urinary bladder may be attributed to individual differences in excretion.

The ability of voxel-level dosimetry in estimating the absorbed dose demonstrated a significant advantage compared to the conventional organ-level method. In the tumor, the absorbed dose estimates were 0.024 $\pm$ 0.003 Gy/MBq, whereas those were not estimated by organ-level dosimetry due to the lack of subject-specific tumor geometry in the MIRD-phantom. Because voxel-level method considers inhomogeneous activity distribution and tissue heterogeneity throughout the entire body, the voxel-level method was expected to yield a more realistic and accurate voxel-level dose distribution in organs, such as the heart, those are consisted of distinguished component.

Furthermore, the differences in the absorbed dose of every organ demonstrated the differences between real mice and virtually designed or phantom mice. Except for the liver and intestine, the absorbed dose estimated by the organ-level method exceeded the voxel-based estimates; the largest difference (0.29 Gy/MBq) was observed for the kidney, albeit in the absence of considering data for real mice.

## Discussion

In response to the rapid growth in the demand for clinically robust estimations of the absorbed dose–response relationship for therapeutic radiopharmaceuticals, PET is being increasingly deployed to characterize the biodistribution, pharmacokinetics, and internal radiation dosimetry of novel companion diagnostic or theranostic radiopharmaceuticals in disease/target-specific xenograft animal models. In this study, we characterized the biodistribution and pharmacokinetics of [^68^Ga]PSMA-11 in PSMA-positive and negative (22Rv1 and PC3, respectively) tumor-bearing mice and subsequently estimated the internal radiation dosimetry via the voxel-level dosimetry method. The use of a dedicated Monte Carlo simulation to evaluate the tumor-absorbed dose directly addresses the drawbacks of the conventional organ-level (or phantom-based) method.

The kidneys and urinary bladder showed substantial accumulation of [^68^Ga]PSMA-11 without exhibiting a washout phase during the period of the study. For the tumor, a peak concentration of 4.5 ± 0.7 %ID/g was recorded 90 min after [^68^Ga]PSMA-11 injection. The biodistribution and pharmacokinetics of [^68^Ga]PSMA-11 in various organs of subcutaneous prostate cancer xenograft model mice were found to be consistent with previously reported data for prostate cancer patients, especially in terms of excretion^11^. Moreover, the pharmacokinetics of [^68^Ga]PSMA-11 were characterized in terms of selectivity and specificity by simultaneously assessing the differences in the level of [^68^Ga]PSMA-11 uptake between PSMA-positive (22Rv1) and negative (PC3) tumors and by assessing the inhibited [^68^Ga]PSMA-11 uptake in the PSMA-positive (22Rv1) tumor following treatment with the PSMA-specific inhibitor 2-PMPA.

Owing to its low molecular weight (< 2000 Da) and hydrophilic nature, [^68^Ga]PSMA-11 clears rapidly from the blood pool, with markedly high uptake by the kidneys resulting from their own high expression of PSMA^12^. This was evident in the biodistribution and pharmacokinetics results, with the rapid peripheral washout from organs involved in systemic and pulmonary circulation contrasting with the substantial accumulation in the kidneys. Radiopharmaceuticals must satisfy several criteria to be considered for clinical cancer diagnosis, such as rapid washout from the background but high and lasting uptake in the target, thereby guaranteeing significant contrast for clear visualization and accurate quantification. The favorable pharmacokinetic characteristics in the tumor, specifically the 5 %ID/g peak concentration (C_max_) 1.5–2 h after administration (T_max_) and the longevity of the uptake in the tumor (T_1/2_), which led to the tumor exhibiting higher accumulation (AUC) than all organs other than the kidneys, define [^68^Ga]PSMA-11 as a good diagnostic radiopharmaceutical for prostate cancer. Our tumor pharmacokinetics data were fairly consistent with previously reported results of prostate cancer patients. The similarity in the biodistribution and pharmacokinetics of [^68^Ga]PSMA-11 between the disease model mice and prostate cancer patients support the reliability of our results for translation to human subjects.

In the present study, we used an open-source Monte Carlo simulation platform, which is called GATE (Geant4 application for emission tomography). GATE was developed by the *OpenGATE Collaboration* in 2004 and based on the commonly used Monte Carlo simulation software Geant4, which provides users with a friendly user interface^13^. Therefore, GATE utilizes many of the advantages of Geant4 when performing dose calculations, including flexible geometric input, voxelized sources and phantoms, a virtual clock capable of simulating time, and various physical models. As such, GATE has been used primarily for studies focused on nuclear medicine imaging, radiation therapy, and dosimetry applications providing personalized dosimetry for targeted radionuclide therapy^9,14–19^. Based on these factors, we selected the GATE Monte Carlo simulation to compute the voxel-level absorbed dose in xenograft model mice after injecting [^68^Ga]PSMA-11. The GATE Monte Carlo simulation is expected to yield a more realistic and accurate voxel-level dose distribution in organs because it considers inhomogeneous activity distribution and tissue heterogeneity throughout the entire body^9^. For example, at organ-level dosimetry, the cumulated activity ($\stackrel{\sim }{\mathrm{A}}$) of blood and the wall of the heart as a source organ, each with different S-values of ^68^Ga, is applied in estimating heart absorbed dose^20,21^. By contrast, the dose maps after direct Monte Carlo simulation are produced considering inhomogeneous activity distribution and tissue heterogeneity from the voxelized source and voxelized phantom. The heart-absorbed dose using dose rate (Gy/s) in all voxels of the heart volumes of interest (VOIs) is estimated in voxel-level dosimetry without distinguishing blood and the wall of the heart. Thus heart-absorbed dose may be expected to overcome the issue of the conventional organ-level (or phantom-based) method.

The level of [^68^Ga]PSMA-11 accumulation corresponded to the absorbed dose in each organ. Using the voxel-level to ascertain dose absorption, the greatest absorbed dose was recorded in the kidneys, measuring 0.209 ± 0.005 Gy/MBq, compared to 0.492 ± 0.059 Gy/MBq estimated via the organ-level method. The differences in the absorbed dose of every organ demonstrated the differences in real mice compared with virtually designed or phantom mice. Statistical differences were recorded in the absorbed doses calculated via the voxel- and organ-level methods for the heart, intestine and kidneys. The ^68^Ga S-values for the walls of the heart and small intestine that were applied in the 25 g mouse model used in this study might also be the reason for such differences^20^, as it was difficult to distinguish heart wall and small intestine accurately during the image-based analysis. In addition, the high accumulation of [^68^Ga]PSMA-11 without washout might be reflected in the self-absorbed dose for the kidney and, if so, would constitute the biggest difference. Moreover, without considering the time-related biodistribution, and the S-values corresponding to non-labeled ^68^Ga radioisotopes may cause discrepancies.

Preclinical dosimetry studies using disease model animals continue to gain interest as molecular imaging is applied in new domains, specifically as a standard theranostic tool for studying the biodistribution and for predicting the clinical radiological safety of novel biomolecules or molecular mechanisms^22^. In particular, it is hoped that preclinical dosimetry can provide a starting point for the radiobiological interpretation and modeling of the dose distribution for response assessment during cancer therapy. For instance, we estimated the effective dose of [^68^Ga]PSMA-11 for humans using normalized residence times converted from the mouse residence times and IDAC/Dose2.1 software according to a method proposed by Garrow et al.^23^. The predicted clinical effective dose was 0.0202 ± 0.0013 mSv/MBq, which is comparable to results reported for human subjects^24^. However, it is unrealistic to calculate the absorbed dose based on tumor-bearing animal models by conventional dosimetry because the pathophysiologic effects between human and animal models differ considerably.

In addition, only a few clinical dosimetry studies have performed the tumor absorbed dose of PSMA targeting PET tracers. Emre Demirci et al. calculated high organ doses in two patients who had bone metastasis^25^. The activity measured from the bone metastasis was added to the activity measured from the rest of the body. Because the MIRD phantoms do not include any tumor tissues, the high calculated data cannot be handled. Further Cho SY et al. determined the absorbed doses to the prostate using the spherical model but they reported tumor biodistribution in SUV_max_ and SUV_mean_ 1 h and 3 h p.i^26^. Hence the preclinical tumor absorbed dose results of prostate cancer xenograft model mice may be meaningful preliminary data because we calculated it by direct GATE Monte Carlo simulation.

As tumors are abnormal anatomical structures, it is difficult to estimate the absorbed dose via the organ-level method without the tumor S-value (S_tumor_) of ^68^Ga. GATE is provided with a *DoseActor* mechanism that stores the absorbed dose in a given volume in a 3D matrix^27^. During particle tracking, the deposited energy is summed in the matrix for each step occurring in the attached volume (${D}_{k}=\sum_{i}^{N}{d}_{k,i}$, N, the number of primary events, ${d}_{k,i}$, the deposited energy in pixel *k* at primary event *i*). The advantages of the GATE-based voxel-level dosimetry method have been demonstrated by its ability to estimate doses of abnormal organs. Therefore, we calculated the tumor-absorbed dose per respective xenograft model mouse using direct Monte Carlo simulation. The voxel-level dosimetry method yielded realistic estimations of tumor-absorbed doses, without being affected by the issues associated with the conventional organ-level method.

To provide a comparison in a tumor, we attempted to estimate the absorbed dose using an alternative approach based on the organ-level dosimetry method embedded in the IDAC/Dose2.1 software assuming that the tumor is a sphere of uniform density and that the distribution of [^68^Ga]PSMA-11 in the tumor is homogeneous regardless of its shape, location, and the target density. The tumor density, tumor volume, and residence time for the real xenograft mice were used as inputs.

Nevertheless, without considering data for real mice, erroneous estimates are likely. As we expected, the absorbed dose (0.079 $\pm$ 0.013 Gy/MBq) was greater than that estimated by voxel-level dosimetry (0.024 $\pm$ 0.003 Gy/MBq). The conditions used for tumor shape and drug distribution are considered to be responsible for the differences in the estimates provided by the two methods. Tumors in mice are ellipsoidal rather than spherical and the tumor uptake is not homogeneous throughout the tumor volume. Furthermore, unlike healthy organs, the tumors in the test mice were inoculated on the side flank, making them much less susceptible to collateral dose absorption from adjacent organs. In healthy organs, the organ-level absorbed dose was commonly overestimated using MIRD schema in comparison with voxel-level dosimetry, because the former assumes homogeneous activity and dose distribution in organs as well as a generalized geometry^28^. Although the application of [^68^Ga]PSMA-11 PET coupled with therapeutic radiopharmaceuticals is currently under broad clinical and scientific investigation, insufficient preclinical studies have been performed to determine the dosing regimen for the therapeutic radiopharmaceuticals. Our data support the use of voxel-level dosimetry in TRT to deliver personalized dosimetry that considers patient-specific heterogeneous tissue compositions and activity distributions.

One limitation of this study is the small number of prostate xenograft model mice (*n* = 3) used in the assessment of the biodistribution and subsequent internal radiation dosimetry experiments. However, unlike in vitro and ex vivo assays of excised organs or tissues, PET measurements allow the minimizing of intra-subject errors between measurements at various time-points, which strengthens the statistical power and allows for efficient data acquisition with reasonably high reliability. To guarantee the reproducibility of the present data, we carefully controlled the experimental factors that may affect inter-subject error, such as the injected radioactivity and purity of [^68^Ga]PSMA-11 (3.10 ± 0.13 MBq and > 99%, respectively) and the tumor environment defined by the size (calipered size = 0.18 ± 0.03 cc). Furthermore, the small-animal dedicated PET/CT system was calibrated on a quarterly basis with the experimental phantom filled with a known concentration of positron emitting radioisotopes, such as ^68^Ga, to guarantee its performance reliability for radioactivity quantification. The analysis was performed in a consistent manner by the expert, which resulted in small errors in the estimates of the parameters of distribution, pharmacokinetics, specificity, and selectivity as well as the absorbed dose.

Voxel-level dosimetry can be applied to estimate the absorbed dose for therapeutic radiopharmaceuticals. By using the image of a surrogate radiopharmaceutical and extrapolating the data preexisting diagnostic drugs, it is possible to indirectly obtain information on the characteristics of the body of a therapeutic radiopharmaceutical that does not emit radiation for imaging. In addition, it is useful for evaluating therapeutic radiopharmaceutical distributions and devising optimized patient-specific treatment plans. Furthermore, patient-specific voxel-based dosimetry has the distinct advantage of being able to predict the biological effects of targeted radionuclide therapy more effectively. Dose-volume histograms (DVHs) can be obtained using the patient-specific three-dimensional distributions of the absorbed dose within the target volume of the tumor, which is particularly useful because it could be used as the starting point for the radiobiologic interpretation and modeling of the dose distribution for response assessment during cancer therapy^29^.

## Materials and methods

### Animals

Male BALB/c mice (6 weeks old) were purchased from Orient Bio (South Korea). All animal experiments were carried out in accordance with the approved guidelines. The study is compliant with the ARRIVE guidelines. All animal experimental protocols were approved by the Institutional Animal Care and Use Committee (IACUC) of Seoul National University Bundang Hospital, Seongnam, Korea. The mice were kept in a specific pathogen-free room maintained at ~ 21 °C and ~ 55% RH on a 12 h light/dark cycle, with food and water available ad libitum before the PET/CT studies. Food for the mice was suspended overnight prior to [^68^Ga]PSMA-11 PET/CT imaging.

#### Subcutaneous prostate cancer xenograft model

PSMA-positive (22Rv1) and negative (PC3) human prostate carcinoma cell lines were purchased from American Type Culture Collection (Rockville, MD, USA). Both cell lines were maintained in RPMI 1640 medium containing 10% fetal bovine serum prior to use.

In total, nine subcutaneous prostate cancer xenograft model mice were generated in-house. Six of the mice had 22Rv1 tumors, which were induced by inoculating 22Rv1 PSMA-positive cells (1.0 × 10^7^ cells in 100 μL phosphate-buffered saline) into the right flank of each mouse. The other three model mice bearing both 22Rv1 and PC3 tumors, which were established by inoculating 22Rv1 PSMA-positive cells (1.0 × 10^7^ cells) into the right femoral of the mice and PC3 PSMA-negative cells (1.0 × 10^7^ cells) into the left femoral of the mice. All the mice were maintained under the abovementioned conditions for approximately two weeks until the tumors reach a size of approximately 3 mm in diameter (measured with calipers). Three 22Rv1 tumor-bearing mice were used in the PET/CT studies to characterize the biodistribution and pharmacokinetics, while the other three 22Rv1 tumor-bearing mice underwent PET/CT studies to investigate the specificity of [^68^Ga]PSMA-11. The three model mice bearing both 22Rv1 and PC3 tumors underwent PET/CT studies to investigate the selectivity of [^68^Ga]PSMA-11.

### Preparation of [^68^Ga]PSMA-11

We used ^68^Ga^3+^ obtained from a ^68^Ge/^68^Ga radionuclide generator (iThemba LABS, Somerset West, South Africa) for the radiolabeling of PSMA-11. The precursor peptides (1 nmol in 0.1 M HEPES buffer, pH 7.5, 90 μL) were added, in a volume of 100 μL, to a mixture comprising 10 μL of 2.1 M HEPES solution and 10 μL of [^68^Ga]Ga^3+^ eluate (50 –100 MBq). Next, the pH of the labeling solution was adjusted to 4.2. Then, the reaction mixture was incubated at 80 °C for 2 min. The radiochemical yield was determined using high-performance liquid chromatography (HPLC). This approach achieved typical radiochemical yields of 52.07 ± 1.42% (non-decay corrected) and radiochemical purity > 99%.

### PET/CT studies

#### Biodistribution and pharmacokinetics

As mentioned above, the biodistribution and pharmacokinetics were studied for three 22Rv1 tumor-bearing mice using an animal-dedicated PET/CT system (NanoPET/CT, Mediso Inc., Budapest, Hungary). Whole-body dynamic PET imaging was performed over a duration of 90 min immediately after administering an intravenous injection of [^68^Ga]PSMA-11 (3.10 ± 0.13 MBq) to each mouse via a catheter inserted in its tail vein. In addition, we acquired PET/CT images at 3, 4, and 5 h after [^68^Ga]PSMA-11 administration. The mice were maintained under 2% isoflurane anesthesia during PET/CT scanning. The dynamic image frames were reconstructed using the iterative three-dimensional ordered subset expectation maximization (OSEM) algorithm and the single-slice rebinning (SSRB) method. During image reconstruction, attenuation corrections were applied for CT-related scatter and decay. The reconstructed images had a volume of 142 × 142 × 163 mm^3^ and a voxel volume of 0.6 × 0.6 × 0.6 mm^3^. A calibration factor was measured from uniform syringe phantoms (3 cc) filled with [^68^Ga]PSMA-11 to correct for the activity concentration (Bq/ml) in the reconstructed PET images. The VOIs were drawn manually over the major organs (tumor, heart, lungs, kidneys, liver, bladder wall and intestine) on the CT and time-integrated PET images using PMOD software (version 3.6, PMOD Technologies Ltd., Zurich, Switzerland), taking care to ensure that the VOIs did not overlap. The number of voxels within the VOIs drawn for an organ at each time point were averaged and multiplied by the voxel volume and tissue density to estimate the organ mass.

The [^68^Ga]PSMA-11 uptake for each organ was estimated for each mouse by applying VOIs over the respective organs on the reconstructed PET images. The PET image-based biodistribution data obtained from the organs were plotted as a function of time to generate time activity curves (TACs). For each organ, the measured activity (in kBq/cc) was normalized to the total injected activity to express the percentage of injected dose per gram (%ID/g).

The pharmacokinetic parameters of [^68^Ga]PSMA-11 in each organ were assessed quantitatively using the TACs of the organs of interest: peak concentration (C_max_), time to reach C_max_ (T_max_), half-life (T_1/2_), and area under the TAC (AUC). The pharmacokinetic parameters were calculated using GraphPad Prism software (version 8.0, GraphPad Software, La Jolla, CA, USA).

#### Selectivity and specificity

To examine the selectivity of [^68^Ga]PSMA-11 by demonstrating the greater uptake of [^68^Ga]PSMA-11 in the PSMA-positive (22Rv1) tumor compared to the PSMA-negative (PC3) tumor, we used the three model mice bearing both 22Rv1 and PC3 tumors. A static 20-min PET/CT study was performed 90 min after the intravenous injection of [^68^Ga]PSMA-11 (2.20 ± 0.38 MBq). To investigate the specificity of [^68^Ga]PSMA-11 by demonstrating the inhibited uptake of [^68^Ga]PSMA-11 in the PSMA-positive (22Rv1) tumor after treatment with the potent and selective inhibitor 2-PMPA (2-(Phosphonomethyl)-pentanedioic acid) of glutamate carboxypeptidase II^30^, we studied the three 22Rv1 tumor-bearing mice before and after 2-PMPA treatment. The mice underwent static 20-min PET/CT studies 90 min after the intravenous injection of [^68^Ga]PSMA-11 (before: 3.10 ± 0.13 MBq; after: 2.87 ± 0.04 MBq).

To compare the %ID/g of [^68^Ga]PSMA-11 in the 22Rv1 and/or PC3 tumors, [^68^Ga]PSMA-11 (2.78 ± 0.02 MBq) was injected into the mice intravenously via the tail vein. We acquired PET/CT image of mice at 60 min after radiotracer injection.

### Internal radiation dosimetry

For this analysis, we used the data acquired from the PET/CT studies for biodistribution and pharmacokinetics.

#### Voxel-level dosimetry method

**Figure 5:**
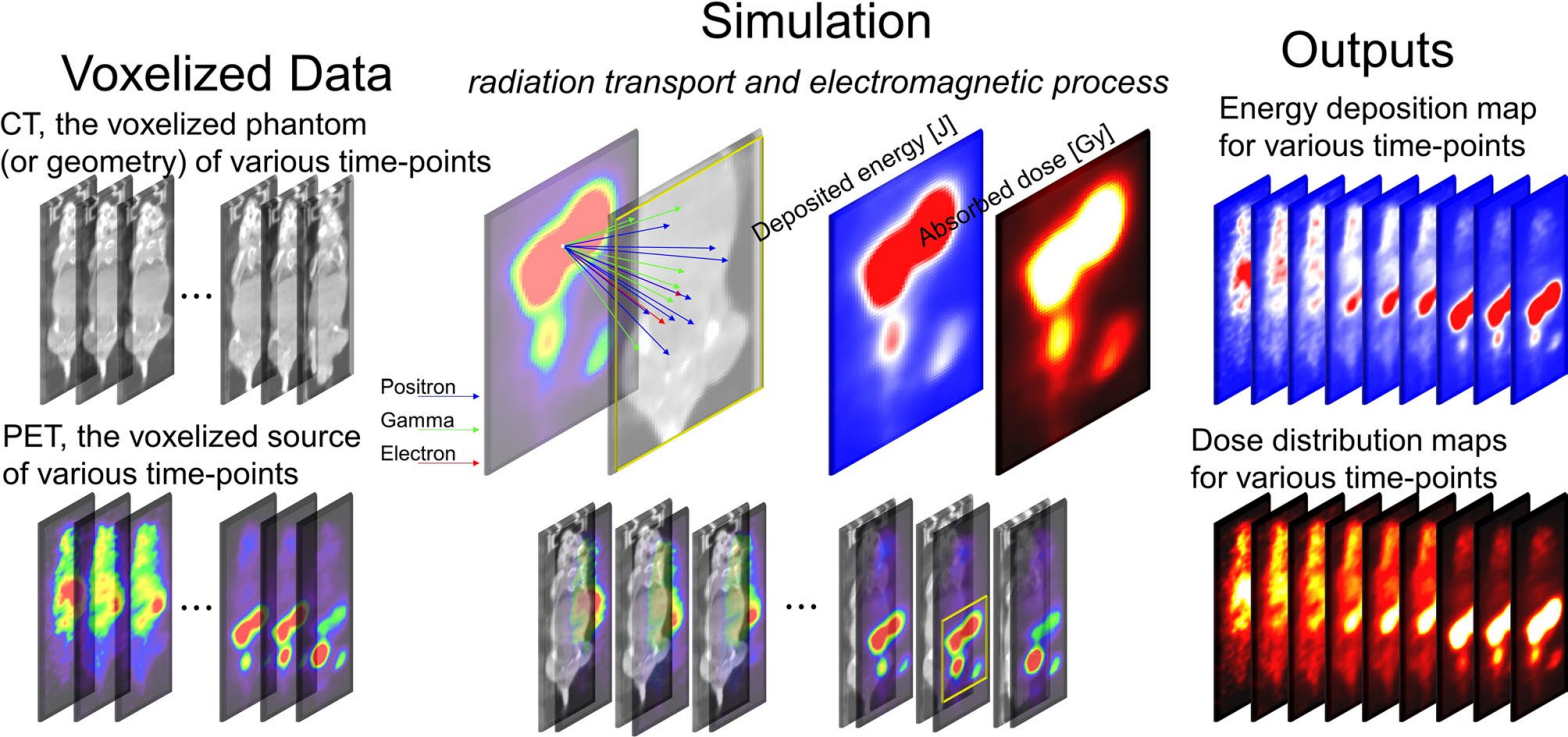
Graphical diagram illustrating how a dose map is generated. GATE simulates electromagnetic physical processes, such as the Photoelectric effect, Compton scattering, Rayleigh scattering, Pair production, Ionization, Bremsstrahlung, Positron and electron annihilation, Single and multiple Scattering and Muon electromagnetic processes for annihilated particles (positron, gamma, electron, etc. depending on the radioisotope) according to the radioactive decay. The trajectories of the particles originate at the specific voxel and the destination is a random voxel. The *DoseActor* mechanism embedded in GATE estimates the deposited energy and the dose map. Finally, the absorbed doses in the voxels are calculated by dividing the deposited energy in each voxel by the voxel mass.

The voxel-based method for estimating the absorbed dose was performed by applying the GATE Monte Carlo simulation (Fig. 5). GATE is based on the Geant4 toolkit^31^, which is a well-established code for radiation transport. All simulations in this study used GATE version 9.0, which has been extended for dosimetry applications. Specifically, GATE contains a mechanism, named *DoseActor*, which stores the absorbed dose in a given volume in a 3D matrix^27^. The CT and PET images of the mice were resampled at the same voxel dimensions (0.6 × 0.6 × 0.6 mm^3^) and used as the voxelized phantom and voxelized source, respectively, representing the inputs to GATE for the dosimetry simulations. The *ImageRegularParametrisedVolume* option was applied for the simulation of a voxelized phantom using the CT image of a real mouse. The ^68^Ga ion-source type from Geant4 version 10.6 was used for the simulation. The standard electromagnetic physics package of GATE, which includes the photoelectric effect, Compton scattering, bremsstrahlung radiation, and positron–electron annihilation, was used for all simulations. Furthermore, the GATE simulation was run using the Mersenne Twister^32^ random number generator. The simulation was run on an in-house computing cluster with a 32-core CPU and 64 GB RAM. For each PET frame, a separate simulation was run based on the corresponding biodistribution data and PET frame durations. The simulation was performed for one-tenth of the acquisition time of each PET frame to reduce the simulation time and computational cost. Nevertheless, the voxel-level statistical uncertainties were kept below 2%^10^. Before conducting the GATE Monte Carlo simulation, we performed validation studies to evaluate the performance, the quantitative accuracy of the ^68^Ga in the virtual soft tissue unit (100 × 100 × 100 mm^3^, 1.04 g/cm^3^), and to set the simulation time.

The simulation outputs the energy deposition (Edep) map, dose distribution map, number of hits, and the local statistical uncertainty. Using the *DoseActor* mechanism, the deposited energy (in MeV) in the voxels within the VOIs drawn over each organ was estimated. Subsequently, the absorbed doses in the voxels were calculated by dividing the deposited energy in each voxel by the voxel mass. Finally, the voxel doses within the VOIs were summed to obtain the absorbed dose for the entire organ. Then, we calculated the dose rate (in Gy/s) for each organ from the PET frame by dividing the absorbed dose by the respective simulation time. The AUC of each dose-rate curve was calculated as the trapezoidal sum of the observed data from 0–300 min and extrapolated to infinity by integrating the physical decay for the curve tail. The voxel-level absorbed dose estimation was normalized to the activity of the injected [^68^Ga]PSMA-11 for each mouse.

#### Organ-level dosimetry method

By applying the MIRD schema^21^, we measured the mean organ-level absorbed dose using the same PET/CT imaging data for the mice to compare the performance of this method against the voxel-based dosimetry estimates obtained via the GATE Monte Carlo simulation. The mean absorbed dose (D) in the target organ (${r}_{t}$) was calculated using the time-integrated activity ($\stackrel{\sim }{\mathrm{A}}$) in the source organs (${r}_{s}$) obtained from the PET image-based biodistribution data and the S-values (S(${r}_{t}$ ← ${r}_{s}$)) using the following equation: D(${r}_{t}$ ← ${r}_{s}$) = $\stackrel{\sim }{\mathrm{A}}$ $\times$ S(${r}_{t}$ ← ${r}_{s}$). For each organ, $\stackrel{\sim }{\mathrm{A}}$ was obtained by calculating the AUC corresponding to the TAC representing the organ. The AUC was calculated as mentioned above for the voxel-dosimetry method. To calculate the absorbed dose in each organ, the S-values of the ^68^Ga radioisotope for the source–target organ pairs were taken from the database published by Xie and Zaidi^20^.

The residence time ($\mathrm{R}$) in the organ was calculated as $\mathrm{R }=\mathrm{ O_{m}}$ $(i)$ $\times$ $\stackrel{\sim }{\mathrm{A}}$ /$A_{inj}$ , where O_m_ is the mass of the organ and * A *_inj_is the administered activity. The human normalized residence times of each radiotracer were obtained from the product of the preclinical residence time of a compartment and a scaling factor, with the latter calculated using (B_r_ / O_r_) $\times$ (O_h _/ B_h_)^23^, where B_r_ and B_h_ are the body masses and O_r_ and O_h_ are the individual organ masses for mice and humans, respectively^33,34^.

### Graphical and statistical analysis

All graphs and statistical analyses were generated using GraphPad Prism 8.0. All quantitative data are expressed as the mean ± the standard error of mean (SEM). The statistical significance was analyzed using the independent t-test. Differences with a *P*-value less than 0.05 were considered statistically significant.
